# Level of physical activity and sleep characteristics in university students

**DOI:** 10.5935/1984-0063.20190092

**Published:** 2019

**Authors:** Félix Arbinaga, Soledad Fernández-Cuenca, Eduardo J. Fernández-Ozcorta, María Dolores Toscano-Hermoso, Miriam Joaquin-Mingorance

**Affiliations:** 1 University of Huelva, Department of Clinical and Experimental Psychology - Huelva - Spain.; 2 School Sport -Group DOGESPORT, Department of Sport - Huelva - Spain.

**Keywords:** Physical Activity, Students, Sleep Hygiene, Nightmares, Sleep Initiation and Maintenance Disorders

## Abstract

**Introduction:**

The aim of the present study was to identify the subjective quality of sleep, the chronotype, the frequency of nightmares and the propensity for nightmares in university students depending on whether they are sufficiently physically active (SPA) or insufficiently physically active (IPA).

**Methods:**

The study involved 855 students (55.67% women) of which 38.01% are IPA. Evaluations were conducted using the Brief Physical Activity Assessment Tool, the Pittsburgh Sleep Quality Index, the Nightmare Frequency Scale, the Nightmare Proneness Scale and The Composite Scale of Morningness.

**Results:**

IPA students showed a greater probability of presenting [OR=2.02] poor sleep quality (> 5 PSQI points) compared with SPA students (*p*<.001). The IPA participants showing a greater probability [OR=3.70] of having an evening chrontoype (*p*<.001), report a higher frequency of nightmares (*p*<.001) and a greater propensity for nightmares (*p*<.001) compared with the SPA participants. In terms of propensity for nightmares an interaction was found between gender and level of PA (F (3,843)=11.360, *p*=.001).

**Conclusion:**

The possibility of implementing strategies to increase the level of PA among university students should be explored, as well as offering sleep hygiene programs that are effective when used in brief sessions.

## INTRODUCTION

An optimal level of Physical Activity (PA) is associated with an improvement in sleep characteristics, when measured both objectively and subjectively^1^^-^3. However, not all of the available data support this relationship^4^. The current recommendations for the practice of healthy physical activity (PA) have been established as: 3 or more sessions per week of 20 min at vigorous intensity or 5 or more sessions per week of 30 min at moderate intensity^5^^,^^6^. Regular exercise has small beneficial effects on total sleep time and sleep efficiency, small-to-medium beneficial effects on sleep onset latency, and moderate beneficial effects on sleep quality.

Such effects appear to be moderated by gender, age, and baseline level of physical activity, as well as exercise type, time of day, duration, and adherence to PA^7^. It has also been shown that the PA interacts with the circadian typology or the chronotype, acting as a synchronizer of the circadian system^8^; there is a significant body of evidence to suggest that exercise may act as a non-photic synchronizer by disrupting circadian rhythms^9^^,^^10^. People with high PA and low levels of sedentary behavior -even if they do not get enough sleep- have greater sleep efficiency and are less tired^1^. Generally, people of the evening chronotype tend to have poorer sleep quality^11^.

Nightmares are also an important aspect of sleep, and appear to be mediated by the level of PA^12^; although this relationship has not always been observed, perhaps due to methodological problems, as some authors have acknowledged^13^. Although research on this issue is scarce, it has been observed that nightmares are more frequent when the level of PA is low^12^. Nightmares can affect the daily functioning of an individual, diminishing their general well-being and sleep quality^14^. Between 2-6% of the adult population report having frequent nightmares (one or more per week) whilst 35-45% of the population report having them at least once a month^12^.

A group that is particularly vulnerable to low PA levels and sleep problems is university students. Between 40 and 50% of university students are physically inactive^15^, with women being less active than men^2^. Previous studies have shown that more than 60% of university students have sleep problems^16^. University students are generally dissatisfied with their sleep, and report having low sleep quality^16^. Similarly, it is reported that university students with a high level of PA have a 49% lower risk of showing poor sleep quality^17^. Further, it has been reported that a high percentage of university students show an evening chronotype^18^. Moreover, there is a tendency for people with an evening chronotype to have poor sleep quality^19^. Accumulating evidence suggests that evening-type adolescents are exposed to a number of determinants that might have a negative impact on their health status. The preference of evening types for delayed bedtimes and rising times is often out of sync with the sleep-wake schedules required by school, work, or social commitments^20^^,^^21^.

Finally, it is worth noting that approximately 5.8% of university students frequently experience nightmares^22^. Those who have nightmares report a greater number of problems related to sleep such as insomnia, difficulty falling asleep, and poor sleep quality^22^.

Taking the background just described as our starting point, the intention was to extend our knowledge regarding the relationship between subjective sleep quality and the level of physical activity in university students. Thus, the aim of the present study was to identify the characteristics related to sleep in university students according to whether they are sufficiently physically active (SPA) or insufficiently physically active (IPA). As working hypotheses we propose: 1. The IPA participants will obtain a higher score on the sleep quality test, which is taken to indicate poor sleep quality; 2. The IPA students have a stronger tendency towards displaying the evening chronotype, and 3. Both the frequency and propensity for nightmares will be higher in the IPA participants.

## METHODS

### Participants

In the present study, 855 university students participated voluntarily (476 women [55.67%] and 379 men [44.33%]), with an average age of 22.55 years (*SD*=4.85). Of the total sample, 38.01% of the participants were classified as IPA (54.6% of the women and 17.20% of the men) whilst 61.87% were classified as SPA (45.4% of the women and 82.8% of the men).

### Instruments

An *ad hoc* interview was used to collect self-report information regarding sex, age, average academic grade, weight, height, and level of physical activity.

The level of physical activity was evaluated with the Brief Physical Activity Assessment Tool (BPAAT)^23^, in its Spanish version^24^. This is composed of two questions that measure the frequency and duration of PA in a “typical” week at different intensities (intense and moderate). The scores are then used to categorize the participants as “SPA” (engaging in > 3 weekly sessions of 20 minutes of high intensity or > 5 sessions per week of 30 minutes of moderate intensity or > 5 sessions of a combination of both intensities) or “IPA” (does not meet the recommended levels of healthy physical activity).

The subjective quality of sleep was assessed using the Pittsburgh Sleep Quality Index (PSQI)^25^ in its Spanish version^26^. The predictive validity of the test is 89.6% for sensitivity and 86.5% for specificity in identifying poor sleep quality with scores greater than 5 points^25^. The Spanish version was shown to have an internal consistency (measured using Cronbach’s α coefficient) of 0.81, whilst the Kappa coefficient was 0.61, with a sensitivity of 88.63%, specificity of 74.99% and a positive predictive value of 80.66^27^. The PSQI is composed of 10 questions, and seven sleep components are analyzed: sleep quality; sleep latency; sleep duration; habitual sleep efficiency; sleep disturbances; use of sleeping medications; and daytime dysfunction due to sleep. A higher score on the PSQI (*Min*: 0, *Max*: 21) is taken to indicate a poor subjective quality of sleep. In the present study, the test showed acceptable internal consistency (α=.77).

To assess the presence of nightmares, two indicators were considered. On the one hand, Item 5h of the PSQI was analyzed: “having problems sleeping due to having nightmares”. On the other hand, the frequency of nightmares was evaluated by using the participants’ estimated number of nightmares. The response options are: 0 = never; 1 = less than once per year; 2 = approximately once per year; 3 = approximately 2 to 4 times per year; 4 = approximately once per month; 5 = approximately 2 - 3 times per month; 6 = approximately once a week; and 7 = several times per week^28^. The test-retest reliability was .75 after four weeks^29^.

The propensity for nightmares was evaluated using the Nightmare Proneness Scale (NPS)^30^. The test showed adequate internal consistency, as revealed by Chronbach’s alpha coefficient (α=.88) and the test-retest correlations were .72^30^. The NPS includes 14 items on which participants respond using a scale ranging from 1 = “Strongly Disagree” to 7 = “Strongly Agree”, obtaining a total score. A higher score is taken to indicate a greater propensity for nightmares. Good internal consistency was obtained in the current sample (α=.848).

Chronotype was evaluated using The Composite Scale of Morningness (CSM)^31^^,^^32^, in its Spanish version^33^. It consists of 13 items that measure the time at which individuals wake up and go to bed, the preferred times for physical and mental activity, and subjective alertness. These data are then used to generate a total score (CSM-Total), (*Min*: 13, *Max*: 55) with a lower score being taken to indicate an evening chronotype, a general morning factor (CSM-General) and an alert factor (CSM-Alert). In particular, those scoring below the 10th percentile are regarded as evening types whilst those scoring above the 90th percentile are regarded as morning types^33^. In this work, acceptable internal consistency was obtained for the CSM-Total (α=.807), the CSM-General (α=.775) and the CSM-Alert (α=.715).

### Procedure

The lecturers of the university were contacted in order to request access to the classrooms and for recruitment of the student volunteers. Volunteers were recruited from years 1, 2, 3, and 4 of the various Degrees of the Faculty (Psychology, Social Education, Primary Education, Infant Education and Sports Science) and in the School of Nursing. The tests were completed during the non-exam period of the course. The investigators supervised the data collection procedure, and the aims of the work were explained to all participants, who were then required to complete the informed consent form. The study was approved by the University Bioethics Committee. All procedures followed were in accordance with the ethical standards of the committee for human experimentation (institutional and national) and the Helsinki Declaration of 1975, as revised in 2000.

### Data analysis

Descriptive statistics are used to show the general characteristics of the sample. Cronbach’s alpha coefficient was applied to determine the internal consistency of the tests. For the quantitative variables, the Student t-test was used, and its corresponding test for effect size (*Cohen’s d*), in which a small effect size is 0.2-0.3; medium effect size is around 0.5; and a large effect size is > 0.8. For categorical variables, the χ^*2*^ test (*df, n*) and its corresponding test for effect size (*Cramer’s Phi or V*) are used. Univariate analysis was conducted in order to determine the interaction between variables. Finally, the Odds Ratio [*OR*] was used to evaluate levels of association according to the conditions considered in each case.

## RESULTS

The male participants in our sample were older than the females (although the effect size is small), and the males also had a greater weight and height than the female participants (Table 1).

**Table 1:** General characteristics of the sample of university students.

	Total (N=855)	Male (N=379-44.33%)	Female (N=476-55.67%)	t	p	Cohen's d
	M (SD)	M (SD)	M (SD)			
Age	22.55 (4.851)	22.97 (4.992)	22.21 (4.713)	2.262	.024	0.16
Weight	65.93 (11.673)	73.27 (9.287)	59.89 (9.837)	20.109	<.001	1.40
Height	169.91 (9.563)	177.56 (6.926)	163.71 (6.388)	30.191	<.001	2.08
Average grade	7.25 (0.769)	7.30 (0.774)	7.21 (0.763)	1.601	.110	
	N (%)	N (%)	N (%)	χ2(1,854)	*p*	Phi
Illness				15.259	<.001	0.13
Yes	158 (18.5)	48 (12.7)	110 (23.2)			
No	695 (81.5)	330 (87.3)	365 (76.8)			
Level of physical activity*				125.199	<.001	0.38
Sufficiently Physically Active	529 (61.87)	313 (82.8)	216 (45.4)			
Insufficiently Physically Active	325 (38.01)	65 (17.2)	260 (54.6)			

* One participant did not complete the Physical Activity Level test.

The women show a greater probability of being IPA (*OR* = 5.80, *95% CI* [4.199-8.001]) compared with men (Table 1).

When the presence of nightmares is examined according to the level of PA (Table 2), it is found that the IPA participants obtain a higher score on the frequency of nightmares scale, the propensity for nightmares, and item 5h of the PSQI, which measures sleeping problems due to nightmares.

**Table 2:** Relationship between the level of physical activity and the frequency and propensity for nightmares, the scores of the CSM, and the PSQI.

	Physical activity level			*t*	*p*	Cohen's d
	Total (N=855)	Sufficiently Physically Active (N=529-61.87%)	Insufficiently Physically Active (N=325-38.01%)			
	M (SD)	M (SD)	M (SD)			
Nightmare frequency	3.50 (1.604)	3.32 (1.613)	3.78 (1.540)	4.100	< .001	0.29
Nightmare propensity	36.99 (12.954)	34.89 (12.585)	40.41 (12.856)	6.137	< .001	0.44
Item 5h PSQI	0.70 (0.786)	0.62 (0.774)	0.82 (0.788)	3.795	< .001	0.26
CSM-Total	32.44 (6.263)	33.22 (6.172)	31.16 (6.220)	4.707	< .001	0.33
CSM-General	24.56 (5.062)	25.15 (4.994)	23.60 (5.038)	4.364	< .001	0.31
CSM-Alert	7.87 (2.080)	8.07 (2.061)	7.54 (2.027)	3.603	< .001	0.26

CSM-Total.- Total score of the Composite Scale of Morningness. CSM-General.- General rating of the composite scale of Morningness. CSM-Alert.- Scores for the Alert factor of the Composite Scale of Morningness.

Similarly, significant differences in chronotype were also found according to the PA level of the students (Table 2). In particular, the IPA participants have lower scores on the CSM-Total, the CSM-General and the CSM-Alert. The IPA students show scores that are closely related to the evening chronotype.

When categorizing the scores on the CSM scale according to evening and morning chronotypes (*evening*: < *10th* percentile [*n*=92 (10.8%)]; *morning*: > *90th* percentile [*n*=102 (11.9%)]) and analyzing the scores according to the level of PA, there are significant differences (χ^2^ (1,194)=18.219, *p*<.001, *Phi*=0.31) between the SPA and the IPA participants, with the latter showing a greater probability of having an evening chronotype (*OR*=3.70, *95% CI* [2.004-6.844]).

Given the relationship between gender and PA level, a univariate factor analysis was conducted between these variables and each of the variables shown in Table 2. This analysis revealed that only the propensity for nightmares is affected by the interaction between gender and PA level (*F(3,843)*=11.360, *p*=.001), which is not the case for the other variables: Frequency of nightmares (*F(3,849)*=3.273, *p*=.071), Item 5h of the PSQI (*F(3,851)*=2.405, *p*=.121), CSM-Total score (*F(3,849)*=0.841, *p*=.359), CSM-General score (*F(3,849)*=0.137, *p*=.712), CSM-Alert score (*F(3,849)*=3.610, *p*=.058), or the total PSQI score (*F(3,853)*=3.478, *p*=.063).

Further, it appears that the level of PA is related to academic performance, with the IPA students (*M*=7.17, *SD*=0.74) obtaining lower academic grades (*t*=2.67, *p*<.001, *d*=0.19) in comparison with the SPA students (*M*=7.31, *SD*=0.78).

Regarding the scores obtained on the subjective quality of sleep tests according to different cut-off points of the PSQI (Table 3), the highest percentage of participants presented a score higher than five on this test, indicating poor sleep quality. There is a tendency (χ^2^ (1,838)=22.971, *p*<.001, *Phi*=0.17) for the IPA students to show a greater probability of having poor subjective sleep quality (>5 PSQI points) (*OR*=2.02, *95% CI* [1.513-2.699]) than the SPA students. There are also differences between the two groups when considering poor sleep quality as scores > 8 on the PSQI (χ^2^ (*1,838)*=19.152, *p*<.001, *Phi*=0.16). In particular, the IPA group shows a higher probability of reporting a poor quality of sleep than the SPA participants (*OR*=2.03, *95% CI* [1.473-2.790]). Similarly, when the adopted criterion for poor sleep quality is scores > 5 in the PSQI (χ^2^ (1,838)=19.293, *p*<.001, *Phi*=0.16), there is a greater probability of the IPA students having poor sleep quality compared with the SPA students (*OR*=2.03, *95% CI* [1.475-2.790]).

**Table 3:** Cut-off points of the Pittsburgh Sleep Quality Index in relation to the level of physical activity.

N (%)	Physical activity level				*p*	Phi
	Total (N=855)	Sufficiently Physically Active (N=529-61.87%)	Insufficiently Physically Active (N=325-38.01%)	χ2(1,838)		
PSQI-6				22.971	< .001	0.17
> 5	474 (56.6)	259 (50.1)	215 (67.0)			
≤ 5	364 (43.4)	258 (49.9)	106 (33.0)			
PSQI-8				19.152	< .001	0.15
> 8	205 (24.5)	100 (19.3)	105 (32.7)			
≤ 8	633 (75.5)	417 (80.7)	216 (67.3)			
PSQI-5				19.293	< .001	0.15
≥ 5	578 (69.0)	328 (63.4)	250 (77.9)			
< 5	260 (31.0)	189 (36.6)	71 (22.1)			

PSQI6 (≤ 5 Indicates good sleep quality; > 5 Indicates bad sleep quality); PSQI8 (≤ 8 Indicates good sleep quality, > 8 Indicates bad sleep quality); PSQI5 (< 5 Indicates good sleep quality, ≥ 5 Indicates bad sleep quality).

Sleep quality also appears to be related to mean scores when measuring academic performance. Those who have poor sleep quality (scores >5 on the PSQI) obtain poorer grades (*M*=7.21, *SD*=0.81) than those who report good sleep quality (*M*=7.33, *SD*=0.69), an effect that reaches significance (*t*=2.116, *p*=.035, *d*=0.15). No interaction was observed between the quality of sleep and the level of PA for academic grades obtained (*F(7,827)*=0.415, *p*=.894).

It was also found that IPA students have higher PSQI scores than the SPA students (*t*=5.022, *p*<.001, *d*=0.35), indicating poorer subjective sleep quality (Table 4). Specifically, for the scores obtained on the seven components of the PSQI (Table 4), the scores were higher for the IPA students compared to the SPA students on six of the components (sleep quality, sleep latency, sleep duration, sleep efficiency, sleep disturbance, and daytime dysfunction due to sleep) indicating poor overall sleep quality in the IPA students.

**Table 4:** Scores of the components of the PSQI according to the level of physical activity.

	Physical activity level			*t*	*p*	Cohen's d
	Total (N=855)	Sufficiently Physically Active (N=529-61.87%)	Insufficiently Physically Active (N=325-38.01%)			
	M (SD)	M (SD)	M (SD)			
PSQI Total	6.55 (3.293)	6.11 (3.206)	7.26 (3.308)	5.022	< .001	0.35
Comp.1-Sleep quality	1.22 (0.751)	1.13 (0.731)	1.35 (0.762)	4.146	< .001	0.30
Comp.2-Sleep latency	1.39 (0.939)	1.31 (0.930)	1.52 (0.941)	3.073	.002	0.23
Comp.3-Sleep duration	0.90 (0.872)	0.85 (0.852)	0.98 (0.893)	2.056	.040	0.15
Comp.4-Sleep efficiency	0.48 (0.789)	0.43 (0.739)	0.58 (0.857)	2.626	.009	0.19
Comp.5-Sleep disturbance	1.15 (0.457)	1.12 (0.452)	1.19 (0.459)	2.247	.025	0.15
Comp.6-Use of medication	0.30 (0.716)	0.27 (0.699)	0.34 (0.743)	1.306	.192	
Comp.7-Daytime dysfunction	1.12 (0.853)	1.00 (0.814)	1.30 (0.877)	5.018	< .001	0.36

PSQI Total.- Total score of Pittsburgh Sleep Quality Index

Grouping into two categories the answers to each of the seven components of the PSQI, and analyzing these according to PA level (Table 5), differences in the quality of sleep were observed. In comparison with the SPA students, the IPA students show a greater probability of reporting poor sleep quality (*OR*=1.81, *95% CI* [1.348-2.420]), greater sleep latency (*OR*=1.43, *95% CI* [1.081-1.886]), and daytime dysfunction due to sleep (*OR*=1.86, *95% CI* [1.386-2.493]).

**Table 5:** Scores of the PSQI components in relation to the Physical Activity Level.

N (%)	Physical activity level			χ2(3,850)	*p*	Phi
	Total (N=855)	Sufficiently Physically Active (N=529-61.87%)	Insufficiently Physically Active (N=325-38.01%)			
^1^Comp.1-Sleep quality				15.872	< .001	0.14
Poor	277 (32.5)	145 (27.5)	132 (40.6)			
Good	576 (67.5)	383 (72.5)	193 (59.4)			
^2^Comp.2-Sleep latency				6.318	.012	0.09
High	380 (44.7)	217 (41.3)	163 (50.2)			
Low	470 (55.3)	308 (58.7)	162 (49.8)			
Comp.3-Sleep duration				3.734	.053	
< 7 hours	115 (13.5)	62 (11.7)	53 (16.4)			
≥ 7 hours	736 (86.5)	466 (88.3)	270 (83.6)			
Comp.4-Sleep efficiency				2.914	.088	
< 75%	98 (11.5)	53 (10.1)	45 (13.9)			
≥ 75%	701 (82.9)	473 (89.9)	278 (86.1)			
^3^Comp.5-Sleep disturbance				1.964	.161	
High	145 (17.1)	82 (15.7)	63 (19.4)			
Low	701 (82.9)	440 (84.3)	261 (80.6)			
Comp.6-Use of medication				1.49	.700	
≥ Once/week	67 (7.9)	40 (7.6)	27 (8.3)			
< Once/week	786 (92.1)	488 (92.4)	298 (91.7)			
^4^Comp.7-Daytime dysfunction				17.340	< .001	0.14
High	274 (32.2)	142 (27.0)	132 (40.7)			
Low	576 (67.8)	384 (73.0)	192 (59.3)			

*1.* -Poor (PSQI categories.- Very bad, pretty bad), Good (PSQI categories.- Pretty good, very good); *2.*- High (PSQI categories.- 31-60 minutes, > 60 minutes), Low (PSQI categories.- < 15 minutes, 16-30 minutes); *3.*- Low (PSQI categories.- Scores between 0 & 1-9), High (PSQI categories.- Scores between 10-18 & 19-27);* 4.*- High (PSQI categories.- 1-2 times per week, > 3 times per week), Low (PSQI categories.- < 1 per week, never in the last month).

## DISCUSSION

The objective of the present study was to analyze how the level of PA in university students is linked to a number of different variables related to sleeping habits. As working hypotheses we proposed that: 1) The score on the sleep quality index will be higher for the IPA participants, which indicates a poorer quality of sleep; 2) The IPA participants will tend to show scores that indicate an evening chronotype; and 3) IPA students will show both a greater nightmare frequency and propensity.

Addressing the first of these hypotheses, the results reveal that the IPA students have higher total scores on the PSQI, which indicates generally poorer sleep quality. This finding is in agreement with other studies that have reported a positive relationship between PA and the quality of sleep in university students^17^. Similarly, IPA students presented higher scores on most of the components of the PSQI, which indicates poorer sleep quality. In particular, these students show greater sleep latencies, consistent with the findings reported by Kakinami et al.^4^; a shorter duration of sleep, in line with previous studies where, after 22 years of age, the hours of sleep decrease^34^; lower sleep efficiency, more sleep disturbances and higher levels of daily dysfunction. The level of PA does not appear to affect the use of sleeping medications.

In relation to the second hypothesis, the present results show that IPA participants have a lower score on the CSM-General, the CSM-Alert the CSM-Total, which indicates a greater tendency towards the evening chronotype. In addition, the evening chronotype group (scores below the 10th percentile) is composed mainly of IPA students. However, in the present study it is not possible to establish a causal relationship between the type of chronotype and physical activity. Therefore it is worth noting that the practice of exercise can act as a non-photic synchronizer, which disrupts circadian rhythms^9^^,^^10^. These results are similar to those found in previous studies^18^, which showed that a significant percentage of the university population are of the evening chonotype. Thus, we should not rule out the possibility that academic demands constitute an environmental factor (e.g., class attendance, study hours) that can facilitate the tendency for an individual to have an evening circardian rhythm^20^^,^^21^.

Regarding the third hypothesis, it was found that the frequency of nightmares is higher in IPA participants, which is in accordance with previous findings^12^. In addition, the propensity for nightmares is also greater in the IPA students. Moreover, in the propensity for nightmares we found an interaction between the gender of the students and the level of PA. Evidence has shown that milder forms of waking life stressors have also been found to trigger the occurrence of nightmares^35^. For example, research has indicated that a stressful event such as exam stress can be the cause of more frequent nightmares^36^.

In general, this study has also provided data that agrees with the findings of previous research on the low level of PA in university students. In our sample, 38.1% are IPA, which is comparable to prevalence rates of 40% found in other studies^15^. In addition, the present findings show that women are more likely to fall under the IPA category, as previously reported^2^.

Among the limitations of this study, it is worth pointing out that the research design used here does not allow for establishing the directionality of causal relationships. Further, assessing PA levels by means of a questionnaire can cause an overvaluation-underestimation of the PA level of the participants. Therefore, in future work objective tests are needed to more accurately evaluate PA levels. Similarly, it would also be interesting to consider the possibility of using tests such as actigraphy in the evaluation of sleep, although subjective evaluations also provide very relevant information.

Finally, the high prevalence of IPA students and those with poor sleep quality make it necessary to consider the possibility of implementing programs aimed at increasing both the level of PA and strategies aimed at improving sleep quality. In this regard, some studies have confirmed the effectiveness and efficiency of behavioral strategies and brief sessions of sleep hygiene, in which sessions lasting only one hour have been shown to be effective in improving the quality and quantity of sleep.
